# Applying real-time Delphi methods: development of a pain management survey in emergency nursing

**DOI:** 10.1186/s12912-021-00661-9

**Published:** 2021-08-18

**Authors:** Wayne Varndell, Margaret Fry, Doug Elliott

**Affiliations:** 1 Prince of Wales Hospital Emergency Department, NSW 2031 Randwick, Australia; 2 Faculty of Health, University of Technology Sydney, NSW 2007 Ultimo, Australia; 3 College of Emergency Nursing Australasia, PO Box 7345, Victoria 3193 Beaumaris, Australia; 4 School of Nursing & Midwifery, Faculty of Health, University of Technology Sydney, 15 Broadway, NSW 2007 Ultimo, Australia; 5 Director Research and Practice Development Nursing and Midwifery Directorate NSLHD, Royal North Shore Hospital, Level 7 Kolling Building, NSW 2065 St Leonards, Australia

**Keywords:** Delphi, Methodology, Consensus, Emergency Nursing, Group Opinion

## Abstract

The modified Delphi technique is widely used to develop consensus on group opinion within health services research. However, digital platforms are offering researchers the capacity to undertake a real-time Delphi, which provides novel opportunities to enhance the process. The aim of this case study is to discuss and reflect on the use of a real-time Delphi method for researchers in emergency nursing and cognate areas of practice. A real-time Delphi method was used to develop a national survey examining knowledge, perceptions and factors influencing pain assessment and management practices among Australian emergency nurses. While designing and completing this real-time Delphi study, a number of areas, emerged that demanded careful consideration and provide guidance to future researchers.

## Background

The Delphi technique is an established and effective research method with multifaceted applications for health services research. The Delphi technique is uniquely designed to explore health issues and topics where minimal information or agreement currently exists, a relatively common situation within nursing practice. Secondly, the Delphi technique allows for the introduction and integration of viewpoints, opinions, and insights from a wide array of expert stakeholders. With increasing accessibility to the Internet and proliferation of smart device technology, changes from paper-based surveys to the development of online software systems, such as the real-time Delphi method, has significantly extended the potential research for the research population and sample, and efficiency of data collection and analysis. However, a recent systematic review highlighted a gap between available methodological guidance and publishing primary research in conducting real-time Delphi studies [1, 2].

In this paper, we seek to examine the methodological gap in applying real-time Delphi methods, by providing a specific case example from a real-time Delphi study conducted to develop a self-reporting survey tool to explore pain management practices of Australian emergency nursing in critically ill adult patients [3]. Insight into the procedural challenges and enablers encountered in conducting a real-time Delphi study are provided. Importantly, key characteristics of the method are presented, followed by the case-based exemplars to illustrate important methodological considerations. Reflections from the case are then presented, along with recommendations for future researchers considering the use of a real-time Delphi technique approach.

### Overview of the Delphi Technique

The Delphi technique was developed in the late 1950s’ by the Research and Development (RAND) Corporation [4] as a method for enabling a group of individuals to collectively address a complex problem, through a structured group communication process without bringing participants together physically [5]. Delphi has value in the healthcare sector, as it is characterised by multi-disciplinary teams and hierarchical structures [6]. The Delphi technique has since become popular with nursing researchers exploring a wide range of topics including role delineation [7–9], priorities for nursing research [10–12], standards of practice [13, 14] and instrument development [15, 16].

The four main characteristics of the classic Delphi method are anonymity, iteration, controlled feedback and statistical aggregation of group responses [17]. Data collection within the classic Delphi typically includes at least two [18] or three [19] rounds of questionnaires facilitated by a moderator. Round one represents what Ziglio [20] termed the ‘exploration phase’, in which the topic is fully explored using broad open-ended questions. Each following round then becomes part of an ‘evaluation phase’, where results of the previous round, interspersed with controlled feedback from a moderator, are used to frame another set of questions. Each round provides an opportunity for expert panel members to respond to and revise their answer in view of the previous responses from other panel members [21]. Since its introduction, over 20 variations of the classic Delphi method have evolved, with researchers modifying the approach to suit their needs. Most common Delphi versions include modified, decision, policy, internet, and more recently real-time Delphi, and have empaneled varying numbers of experts ranging from 6 to 1,142 [22, 23] (Table 1).

**Table 1:** Common types of Delphi and their key differences

Delphi technique, description	Anonymity	Iterative process	Feedback	Statistical aggregation
**Classic Delphi**: generate ideas, elicit opinions and gain consensus on a given topic [4]	Maintained	• Series of rounds • Round 1 commences with an open-ended questionnaire, with subsequent phases used to evaluate responses	• Controlled feedback by moderator between each round	• At conclusion of the final round
**Modified Delphi**: similar process as the classic Delphi, modifications commonly alter round 1, or facilitate contact between panelists [24, 25]	Variable	• Series of rounds • Modifications typically take the form of replacing round 1 (exploratory phase) with pre-generated items from the literature, or replacing round 1 with face-to-face interviews/focus groups	• Controlled feedback by moderator between each round	• At conclusion of the final round
**Decision Delphi**: same process as classic Delphi, however purpose is to formulate, assist or make decisions, as opposed to coming to a consensus [24]	Maintained	• Series of rounds • Round 1 commences with an open-ended questionnaire	• Controlled feedback by moderator between each questionnaire	• At conclusion of the final round
**Policy Delphi**: follows classic Delphi process, focus is to elicit breadth of views and opinions, both common and divergent, on policy issues, and come to a consensus on future policy [26]	Maintained	• Series of rounds • Round 1 commences with an open-ended questionnaire	• Controlled feedback by moderator between each questionnaire	• At conclusion of the final round
**Internet Delphi**: same processes as the classic Delphi, conducted using an online platform [27]	Maintained	• Series of rounds • Round 1 commences with an open-ended questionnaire, with subsequent phases used to evaluate responses	• Controlled feedback by moderator between each questionnaire	• At conclusion of the final round
**Real-time Delphi**: similar process as the classic Delphi, uses special software to conduct a ‘round-less’ real-time survey of experts to generate consensus [28]	Maintained	• No rounds, single questionnaire used • Experts can access the system throughout a set time period, review, comment and revise their assessments as needed	• When a panelist assess a statement they are immediately confronted with the aggregated results (quantitative and qualitative) of all other experts’ estimations	• Continuously updated in real-time until end of study timeframe

The ubiquitous and interactive capacity of the Internet and smart device technology offers benefits that are intimately linked with contemporary research innovations in healthcare [24]. Two clear limitations of the classic Delphi technique were prolonged study durations and high panel member attrition [25]. Aiming to overcome these issues, Gordon and Pease [26] developed the concept of an information technology-enabled contemporaneous extension called real-time Delphi, to improve speed of the data collection process and syntheses of opinions. Conducting a real-time Delphi relies on specially designed software to administer the survey; the functionality or capabilities of which can negatively impact on the success of a study. Initial thoughts of using technology to facilitate the Delphi process emerged as early as 1975 [27]. The first specifically designed real-time Delphi software was developed in 1998 called Professional Delphi Scan [28], with the first real-time Delphi surveys performed and published in the early 2000 s [29]. Since then, several real-time software-based tools have been developed, often by researchers for the purposes of their study [30–32]. However, these have not been evaluated in detail in the literature.

**Fig. 1:**
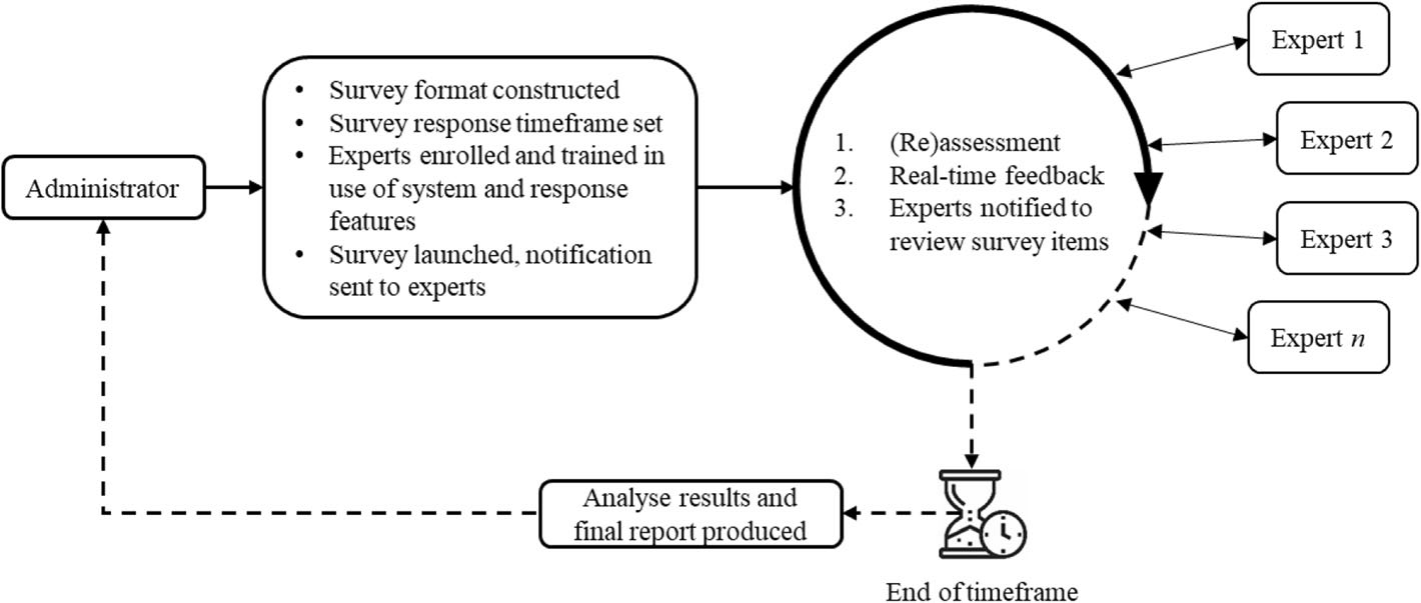
Real-time Delphi processes

In a real-time Delphi process, participants are provided with access to an online questionnaire portal for a specific amount of time. On accessing the portal, expert panel members see all their responses to items and the ongoing, hence real-time, anonymised responses from other panel members. The core innovation of real-time Delphi studies is the simultaneous calculation and feedback. Unlike the classic method, in a real-time Delphi participants do not judge at discrete intervals (i.e. rounds), but can change their opinion as often as they like within the set timeframe [33] (Fig. 1).

## Method

### A real-time Delphi case exemplar

A real-time Delphi study was conducted to develop a context specific instrument (i.e. survey) to investigate emergency nurses’ practices in managing acute pain in critically ill adult patients. The following steps were followed in designing and conducting our real-time Delphi study: study design, pilot testing, recruiting experts, retention, data analysis and reporting. Findings from this study are reported elsewhere [34]. The real-time Delphi method was selected to: maximise participation from expert panel members geographically separated, minimise the amount of time demanded of experts, enable equal flow of information to and from all members, real-time presentation of results to enable experts to reassess and adjust their opinion, and allow panel members a greater degree of expression [35, 36]. Prior to commencing the study, a comprehensive literature review was conducted by the research team to generate initial survey items, and used the following questions:

- What indicators would signify that acute pain in the critically ill adult patient has or has not been adequately detected?
- What indicators would imply that acute pain in the critically ill adult patient has or has not been adequately managed?
- What indicators would suggest that acute pain in the critically ill adult patient has or has not been communicated adequately?

A total of 74 items were initially generated from the literature, and organised into six domains: clinical environment, clinical governance, practice, knowledge, beliefs and values, and perception. Next, commercially available real-time Delphi survey systems were evaluated for their suitability. This process was guided by reviewing the literature [33], trialing available platforms and examining fee structures. Following this review, Surveylet (Calibrum Inc., Utah) was selected [37]. Survey items were then uploaded into Surveylet software system. Pilot testing was then conducted by the research team to evaluate software settings, automation, flow and ease of navigation. Average time to complete the survey was 38 min (SD 8 min).

An expert panel size of 12 to 15 was selected. Identification and selection of experts occurred in three stages: defining the relevant expertise, identifying individuals with desired knowledge and experience, and retaining panel members. First, a pro forma listing the type of skills, experience, qualifications, relevant professional memberships and academic outputs (e.g. peer-reviewed publications) as traits of a desired expert panel member. Second, the research team added potential experts to the list: names of academics were identified via a review of the pertinent literature, with emergency nursing clinicians identified from contacting the College of Emergency Nursing Australasia. Third, initial contacts were approached and provided with a brief overview of the study; pertinent biographical information was then obtained. In addition, they were invited to nominate other experts to be approached for inclusion. Contacts were then independently ranked, with the top 15 experts invited to participate. Twelve accepted the invitation to participate: eight emergency nurses, most nurse consultants (n = 6), two pain management nurse consultants, and two emergency nursing academics from across Australia with an average of 18 years clinical experience. All experts held postgraduate qualifications and half had published in emergency nursing practice and/or pain management.

In Delphi studies, an *a priori* level of consensus and stability sought for the items the experts will rate is set by the research team. In this study, consensus was achieved if ≥ 83 % (10 out of 12 panel members) of experts ranked the item ≥ 7 on the 9-point Likert scale. Secondary measures of consensus among experts included stability of response, evaluated using coefficient of quartile variation (< 5 %) and interclass correlations (≥ 0.75) [38, 39]. Items were retained if primary and secondary measures were met. Data were analysed using median, range and interquartile range. Descriptive statistics were then developed in tabular form and scatterplots.

**Fig. 2:**
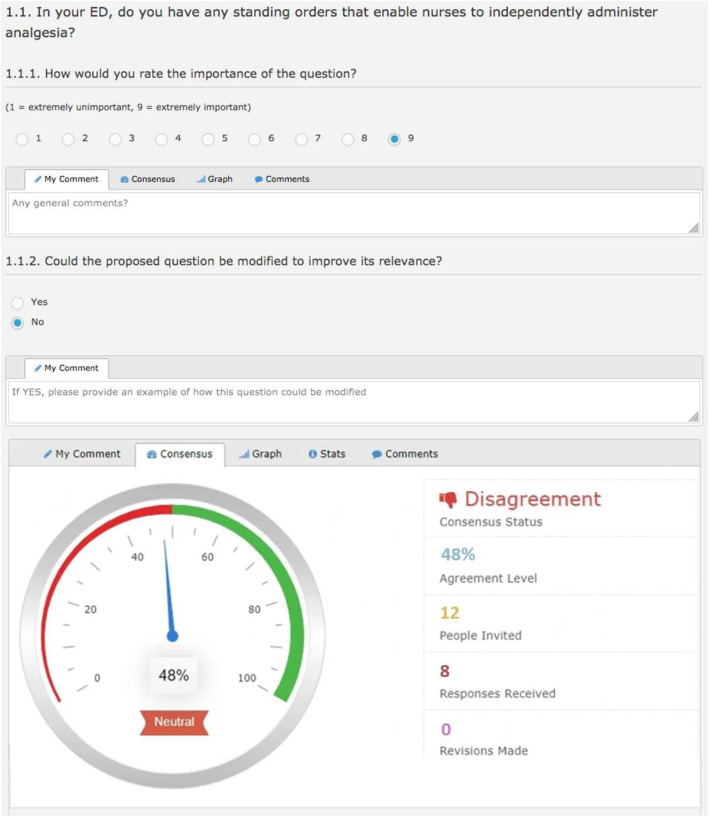
Example of review screen

The Delphi panel members were introduced to each survey domain as they navigated through the real-time Delphi software system, including descriptions of the domain and response format to rate each item. For ease of navigation, one item was presented per page, which included a real-time statistical summary and anonymised remarks from other experts (Fig. 2) [37].

Experts were asked to rate the importance of each question using a 9-point Likert scale (1, extremely unimportant to 9, extremely important), and whether the question could be modified to improve its relevance (Yes/No). If modifications were suggested, respondents were able to provide an example of how the proposed question could be revised, which could then be subsequently voted on by the expert panel.

Participation was asynchronous with experts able to independently re-visit the real-time Delphi survey portal and modify their responses at any point in time between 1st February and March 14th 2019 (a total of 35 days). On accessing the survey portal, panel members can engage in the consensus process from the outset by viewing other panel members’ have responded. Panel members could view not only their own quantitative responses but also the median, range and interquartile range of all given quantitative responses. In the same way, panel members could also view all qualitative arguments submitted by panel members including their own. Panel members could then review or change any or all their responses, or add new arguments, up until the survey closed. Prior to launching the Delphi study, the research team piloted accessing the survey portal, data collection and analysis methods.

## Results

All panel members participated in the survey, providing on average four responses per survey item. Further, of the 74 items initially proposed, 58 (78.4 %) reached consensus in the first week of the study commencing. Following feedback from the expert panel, of the initial 74 items proposed, 12 (16.2 %) were modified to improve clarity, and a further 17 items were added by the expert panel to improve survey depth. At the conclusion of the real-time Delphi, the final survey contained 91 items.

While completing the real-time Delphi, several areas were identified as needing consideration when using this technique: software selection, rating scale, piloting, recruiting experts, consensus and stability, retention and reporting. Key issues are discussed in the following section.

## Discussion

### Software and survey design

This case study has highlighted that to conduct a real-time Delphi requires specialised software. A recent review [33] independently evaluated the characteristics of four commercially available real-time Delphi software solutions (Risk Assessment and Horizon Scanning, eDelfoi, Global Futures Intelligence System and Surveylet) for their range of features and available question formats; data analytics; user friendliness; and, intuitive system operation (Table 2). Surveylet (Calibrum Inc., Utah) [37] was rated the highest for its flexibility, breadth of inbuilt data management options, anonymity of participants and security. While the Surveylet system can amply conduct a real-time Delphi, the system is sophisticated and requires additional time and guidance to configure correctly. Training is provided by way of video tutorials, and support options are available to assist in survey setup at an additional cost. We recommend that prior to conducting a real-time Delphi, researchers comprehensively review, and where possible, trial available software solutions.

**Table 2:** Comparison of Real-time Delphi software system limitations

Real-time Delphi software system	Summary of key limitations
Risk Assessment and Horizon Scanning (RAHS)	• No ability to alter survey layout • No pre-test feature available • Structure of survey limits understanding of real-time • No user manual / technical resources provided • Partial respondent anonymity
eDelfoi	• Limited ability to alter survey layout • No pre-test feature available • Real-time factor is not represented to participants in an untestable way • Software system is unstable, technical issues limit use of software system
Global Features Intelligence System (GFIS)	• Limited ability to alter survey layout • Real-time factor is not represented to participants in an untestable way • Limited data output compatibility • Software system is unstable, technical issues limit use of software system • Software system is complex and is not intuitive • Respondent anonymity not guaranteed
Surveylet	• Software system is comprehensible, but some features require more explanation

Advantages of conducting an online Delphi study include: reduced data entry errors due to automated entry, fewer instances of panelists missing questions resulting in incomplete data, length of time decreases for data collection, and automated aggregation of results and feedback to panelists [40]. The principle difference between a conventional online Delphi and real-time Delphi software systems is the immediate calculation and provision of group responses, which can assist in generating time-sensitive guidance. While there are advantages (Table 3), there are also challenges, which are principally associated with software complexity [35] and cost [41].

**Table 3:** Strengths and challenges of the real-time Delphi

Strengths	Challenges
• ‘Round-less’ • Anonymity, controlled feedback and group response preserved • High efficiency; shorter timeframe required to perform analysis • Immediate calculation and distribution of participant’s responses • Asynchronous participation [42] • Applicability to a wide range of health-related time-sensitive issues • Web-based software; participant materials can be easily shared via hyperlinks embedded into survey • Automation; reduced administration intervention required • Participants can interact and revise their response as often as they want; increased cognitive examination • Participants can immediately interact with each other’s responses • Analysis of quantitative (rating) and qualitative (justification) strengthens participant’s ability to recapture their own point of view, and increases validity of the consensus [43] • Potential to invite large numbers of participants across a wide geographical area	• Requires specially designed software • Internet connection required to access real-time Delphi software platform if web-based • Cost may increase e.g. support to configure survey, duration of hosting survey, number of participants enrolled • Configuring survey can be complicated • System navigation may be difficult for those unfamiliar with the software platform

Internet accessibility, system navigation difficulties and the inconvenience of entering data into a computer-based data screen are recognised as challenges [44]. While the internet is a tool for extending the potential research population and sample, navigating an unfamiliar virtual landscape may frustrate panel members and therefore limit the number of completed surveys [45]. To minimise potential software complexity issues in our study, panel members were sent detailed written instructions on how to access and navigate the real-time Delphi software system, and could attend a one-to-one videoconference with a member of the research team to assist in using the platform [17, 46]. Cost-efficiency is often stated as a key benefit of using online survey tools to conduct an electronic survey [41]. However, in our review of commercially available real-time Delphi software systems, we found that it can become expensive with system providers potentially charging per survey, the number of system administrators or participants enrolled, the duration of the survey, and/or system support to aid survey customisation. While further evidence is needed to substantiate the claim concerning the efficiency of the real-time Delphi method compared to multi-round Delphi designs, current multi-round Delphi studies investigating topics relating to emergency nursing practice, have taken 60 [43] to 273 [13] days to complete.

### Rating scale

Currently, there is no agreement about what rating scale size should be used in Delphi studies; despite being a common reason cited for study failure [47–49]. Rating scales used in previous Delphi studies exploring aspects of emergency nursing practice have ranged from 4 to 11 [1]. While 5 and 7-point scales are the most common forms of Likert scales used in surveys [50, 51], 9-point Likert scales are frequently used in Delphi studies, particularly during the consensus process [47, 49, 52]. A wide range of Likert rating scale sizes can be set within Surveylet. In addition, to underline their rating, experts can also detail their reasoning behind their selection, which can be viewed by other panel members. According to Best [53], accuracy can be improved if experts are provided with both quantitative and qualitative arguments. In a real-time Delphi experts are able to immediately react on each other’s responses, increasing the degree of information experts can interact with, which may aid in recapturing their own point of view [26].

### Piloting

Despite the administrative complexity of conducting any Delphi method, there is limited discussion on pilot testing in the literature. Pilot testing can be conducted to test and adjust the Delphi survey to improve comprehension [54]. When using online software, such as when conducting a real-time Delphi, to conduct and collect multiple responses, the potential impact on cost, time, participant motivation and data integrity should an error occur, could jeopardise the overall study. Pilot testing is therefore vital to identify potential technical or system configuration errors, data collection irregularities (i.e. logic settings) and strengthen participant orientation, prior to commencing the study [55]. Prior to initiating the real-time Delphi, we first verified system configuration and all settings (e.g. timeframe, communication templates), panel member contact details, and that survey items were uploaded correctly. Second, members of the research team independently piloted the survey as mock participants and system administrators, to evaluate the survey flow and ease of navigation. Average time to complete the survey was 38 min (SD 8 min).

### Recruiting experts

The formulation of an expert panel and its makeup is of critical importance for all Delphi studies, yet raises methodological concerns that can negatively impact on the quality of the results [36, 56, 57]. Despite criticism in the literature about Delphi as a methodological approach [2, 17, 35, 36, 40, 58], there remains little agreement as to what defines an expert [36]. Keeney et al. [59] in their review identified several definitions of ‘expert’ ranging from someone who has knowledge about a specific topic, recognised as a specialist in the field, to an informed individual. A recent systematic review of the Delphi method in emergency nursing [1] found similar emphasis in the criteria commonly used to identify experts: length of clinical experience, professional role (e.g. educator, clinical nurse consultant), professional college membership, peer-reviewed publications and postgraduate qualifications. From the current literature, it suggests that defining who is an expert may not be about the role they occupy, but what attributes they possess: knowledge and experience [36, 59–61].

Recruiting experts in our study required: defining the relevant expertise, identifying individuals with desired knowledge and experience, and retaining panel members. Melynk et al. [62] suggests that a minimum threshold for participation as an expert on a Delphi panel should include those measurable characteristics that each participant group would acknowledge as those defining expertise, appropriate to the context, scope and aims of the particular study. While selection of panel experts in Delphi studies typically involves non-probability sampling techniques, which potentially reduces representativeness [17, 57], the aim of our study was to recruit academic and emergency nurses with knowledge and clinical experience in the phenomena being explored – pain management practices for adult critically ill patients [55]. To achieve this, the procedure detailed by Delbecq et al. [63] was followed.

### Expert panel size

Presently there is no agreement in the literature concerning expert panel size [2]. A recent review of 22 Delphi studies within emergency nursing reported a wide range of panel sizes - from fewer than 12 up to 315. Duffield [64] suggests that when a Delphi panel is homogenous 10 to 15 people are adequate. In a similar Delphi study seeking to develop a self-completed survey to examine triage practice, 12 experts were recruited [16]. As noted earlier, the target panel size in our study was 12 to 15, however, as Hartman and Baldwin [65] highlight, due the higher degree of automation of real-time Delphi software systems, typically web-based, the number of experts over a large geographic area participating in a real-time study can be increased.

### Retention

Keeping participants fully engaged once recruited is challenging [40, 57]. High attrition rates can negatively impact on the clarity and validity of results (i.e. item consensus and selection) [56]. Conducting a classic multi-round Delphi study can be a slower process with respect to receiving and analysing feedback, generating the next survey round and determining consensus, and potentially increases the risk of attrition [66]. A potential benefit of the real-time Delphi is its expediency [67]. The much shorter timeframe between panel members submitting their response and getting insights into others’ responses, encourages stronger cognitive examination with the respective issue in question; maximising the validity of results [65]. Presently there is no formal guidance within the literature as to what constitutes an appropriate timeframe with regards to the real-time Delphi method. However, consideration should be given to the overall consensus process timeframe, to ensure panel members have sufficient time to explore opinions to minimise the potential risk of acquiescence bias. To detect potential acquiescence bias, dispersion measures such as range and coefficient of quartile variation were used.

As noted by Zipfinger [42], asynchronous participation can also aid in retaining panel members. Panel members are able to access the Delphi portal at any time, 24-hours a day within the set timeframe, making it more convenient to participate and review feedback. Further, panelists can contribute to whatever aspects in the survey they want, especially when having gone through each question at least once [68].

To maintain panel member engagement, we employed a variety of methods, beginning with participant information sheets. Information sheets were designed based on recommendations from the literature [58, 69], to ensure straightforward messaging on the importance and appeal of the study, aims, processes, timeframe, and benefits, all in clearly marked subsections. To further encourage potential experts who may have had little experience in participating in a real-time Delphi study, we detailed how participants would be introduced to the study, the Delphi methodology, availability of one-on-one training sessions in the use of real-time Delphi software system, and access to technical support. Once the study commenced, the real-time Delphi software sent personalised reminder emails at weekly intervals to encourage participants to (re)assess items in a timely fashion, and provided a summary of responses received to date. These emails emphasised that their views mattered and that for the results to be meaningful, it was important to complete the Delphi process. Sending reminder emails once the Delphi has commenced, can potentially increase retention and response activity of experts [58, 70]. However, while a recent study examining the experiences of Delphi participants concluded that receiving reminders to participate where not viewed negatively, it did not explore frequency [71]. At the completion of the real-time Delphi, panel members were sent a certificate thanking them for their commitment to the study [58], and to provide evidence for their professional development records [72]. Within our study, the level of response activity appears to suggest retention and engagement strategies were effective.

### Consensus and stability

Quantifying the degree of consensus among experts is an important element of Delphi data analysis and interpretation, however reaching a pre-calculated threshold value (e.g. greater than 80 %) of consensus is not the general aim, and rarely is it 100 % [73, 74]. Consensus can either be used to determine if agreement exists or as a stopping guideline, and is measured at the conclusion of a preset number of rounds [75]. Further, as previous studies have demonstrated [49, 76], results can be greatly impacted by the level of consensus set and rating scale used [48, 77]. Within the emergency nursing literature, consensus thresholds have ranged from 50 % [78] to 90 % [79]. Our study used the most common consensus level from previous Delphi studies that had identified survey items as being essential when rated by at least 80 % of the experts [1].

Stability of consensus is also important, which is best evaluated using measures of dispersion [54, 67]. Assessing stability can occur between consecutive rounds, such as in the classic Delphi, or at the conclusion end of the consensus process. While the use of mean, standard deviation and parametric statistics to describe ordinal data is not strictly incorrect when the data is not irregular [80, 81], the use of median, range and interquartile range based on Likert-type scales is favoured as they are more robust to being sensitive to outliers [57]. In classic Delphi, stability is judged between rounds. In real-time Delphi, stability of response is evaluated at the end of the study. In our study, a coefficient of quartile variation (CQV) value less than 5 % was set *a priori* [82], and configured in Surveylet as a measure of relative dispersion based on interquartile range. It is also a measure of homogeneity (i.e. internal consistency) appropriate for small sample (i.e. panel) sizes of 15 or less [83], expressed as:

$$ CQV=\left(\frac{Q_3-{Q}_1}{Q_1-{Q}_3}\right)\kern0.5em \times \kern0.5em 100 $$

In addition, interclass correlations were calculated to inferentially determine stability (≥ 0.75) of responses [38, 39]. Descriptive statistics were then developed in tabular form and scatterplots. Survey items that met the above consensus and stability criteria were incorporated into the final survey (Table 4).

**Table 4:** Example of consensus and convergence amongst experts

Question	Domain	Consensus (%)	Median (Range)	CQV (%)	ICC (95 % CI)
Patient’s families often communicate to me their desire for pain relief for their relative (the patient).	Practice	94.4	8.50 (7–9)	2.7	0.84 (0.79-0.91)
Are current nurse-initiated analgesic standing orders sufficient to manage acute pain in patients presenting to ED?	Governance	95.9	8.75 (8–9)	1.1	0.91 (0.88-0.94)

*CQV* Coefficient of Quartile Variation; *ICC* Interclass Correlation Coefficient

### Reporting

Regardless of what Delphi study design and approach is adopted, attention to rigour of reporting throughout the process is a vital aspect of research. Trustworthiness of the Delphi technique has been debated in the general and nursing literature. Keeney et al. [36] and Powell [84] suggest that the Delphi technique should not be judged by psychometric criteria used for more positivist approaches, with several criteria proposed to evaluate trustworthiness of qualitative studies [55, 85–88]. A common purpose among criteria is to support trustworthiness by reporting the process of study design and data analysis accurately.

In our study, we elected to apply the criteria proposed by Lincoln and Guba [86], based on four concepts; credibility, transferability, dependability and confirmability. Our real time Delphi was based on consensus amongst experienced individuals familiar with the phenomena being explored, across emergency nursing, pain management and academia (credibility and confirmability). Decisions on development of survey questions was arrived at through a documented and auditable; a processes supported by the Surveylet software system (credibility and dependability). The anonymous and continuous process of real-time Delphi research fostered honesty and verification of panelist responses, as panelists could provide feedback and ‘member-checking’ without fear of reprisal from their colleagues (credibility) [36]. Prior to initiating the real-time Delphi process, we piloted the survey for its structure, flow, ease of navigation and robustness (transferability).

## Conclusions

Many papers describe the use of the classic Delphi approach in health services research, yet few provide practical advice on the type and process for undertaken such a research design using the real-time Delphi method. This article presented a case exemplar of a real-time Delphi study and the development of a survey to explore emergency nursing practice. The real-time Delphi method can be of great use in a wide range of time-sensitive health research issues where divergent opinion or little agreement exists. Our experiences have highlighted important strengths and challenges in its deployment, including several methodological issues which may provide guidance to other researchers.

## Data Availability

Not applicable.

## Abbreviations

CQV: Coefficient of Quartile Variation.
RAND: Research and Development Corporation.
SD: Standard Deviation.
